# The Scale-Free Dynamics of Eukaryotic Cells

**DOI:** 10.1371/journal.pone.0003624

**Published:** 2008-11-04

**Authors:** Miguel A. Aon, Marc R. Roussel, Sonia Cortassa, Brian O'Rourke, Douglas B. Murray, Manfred Beckmann, David Lloyd

**Affiliations:** 1 The Johns Hopkins University Institute of Molecular Cardiobiology, Baltimore, Maryland, United States of America; 2 Department of Chemistry and Biochemistry, University of Lethbridge, Lethbridge, Alberta, Canada; 3 Institute for Advanced Biosciences, Keio University, Tsuruoka City, Yamagata, Japan; 4 Institute of Biological Sciences, University of Wales, Aberystwyth, Wales, United Kingdom; 5 Microbiology Group, Cardiff School of Biosciences, Cardiff University, Cardiff, Wales, United Kingdom; Tel Aviv University, Israel

## Abstract

Temporal organization of biological processes requires massively parallel processing on a synchronized time-base. We analyzed time-series data obtained from the bioenergetic oscillatory outputs of *Saccharomyces cerevisiae* and isolated cardiomyocytes utilizing Relative Dispersional (RDA) and Power Spectral (PSA) analyses. These analyses revealed broad frequency distributions and evidence for long-term memory in the observed dynamics. Moreover RDA and PSA showed that the bioenergetic dynamics in both systems show fractal scaling over at least 3 orders of magnitude, and that this scaling obeys an inverse power law. Therefore we conclude that in *S. cerevisiae* and cardiomyocytes the dynamics are scale-free *in vivo*. Applying RDA and PSA to data generated from an *in silico* model of mitochondrial function indicated that in yeast and cardiomyocytes the underlying mechanisms regulating the scale-free behavior are similar. We validated this finding *in vivo* using single cells, and attenuating the activity of the mitochondrial inner membrane anion channel with 4-chlorodiazepam to show that the oscillation of NAD(P)H and reactive oxygen species (ROS) can be abated in these two evolutionarily distant species. Taken together these data strongly support our hypothesis that the generation of ROS, coupled to redox cycling, driven by cytoplasmic and mitochondrial processes, are at the core of the observed rhythmicity and scale-free dynamics. We argue that the operation of scale-free bioenergetic dynamics plays a fundamental role to integrate cellular function, while providing a framework for robust, yet flexible, responses to the environment.

## Introduction

In their long evolutionary history, unicellular and multicellular organisms have pursued the two divergent, although complementary, goals of matching the time dependencies of their internal environments with the periodicities of the external world (i.e. the elaboration of annual, seasonal, daily and tidal rhythms), and optimizing for tolerance to external perturbation [1], [2], [3], [4], [5]. As a result, living systems have developed rhythms that provide internal coordination to maintain spatial and temporal organization from the microscopic to the macroscopic levels [1], [4], [6], [7], [8], [9]. For instance, the provision of energy, biosynthetic pathways, assembly of multimeric proteins, membranes and organelles, stress responses, cell differentiation, migration and cell division require temporal organization on many time scales simultaneously [10], [11], [12], [13]. This complex biological timing requires more than circadian organization; coordination on the ultradian domain (i.e. faster time scales where clocks cycle many times in a day) is essential. Examined more closely, it is evident that additional clocks are required, for instance, a circahoralian clock provides a time base on a scale of hours [14] while faster rhythms or oscillations measured in minutes [15], [16], seconds [17] or milliseconds [18] abound in biological systems. This leads to the central but enigmatic questions in biological timekeeping of whether synchrony occurs between these disparate oscillators, and how function correlates across different time domains.

The concept of scaling [11], [19], [20], [21], [22] provides a theoretical basis to answer questions about interactions and correlations across different spatio-temporal domains. These theoretical concepts as applied to the topological architecture of different kinds of networks showed their non-random scaling properties [23], [24]. Geometric and dynamic fractals have been successfully used to describe spatial and temporal correlations, respectively, across many levels of organization, owing to their intrinsic self-similarity and scaling properties [21], [25]. A recent example is given by the analysis of mitochondrial membrane potential (ΔΨ_m_) noise and the spatial organization of the cardiac mitochondrial network [18]. These findings, which complement other observations of the scale invariance of cardiac function [26], [27], [28], provide fundamental insights relevant to describing and diagnosing pathological conditions [29], [30], [31]. Similarly, synchronization of multioscillatory states, implying controlled chaotic behavior of selected orbits [32], [33], [34] appear to be essential properties governing the coordination of metabolism and transcription across a population of single celled organisms, as shown for continuous cultures of *S. cerevisiae* [13], [35].

Hence, in the present work, we sought to determine if there are commonalities in the biological organization and fractal scaling of bioenergetics in two evolutionarily distant cellular systems, i.e., bakers yeast and cardiomyocytes, utilizing precisely defined physiological and pathological conditions. Using a combined experimental and computational approach we demonstrate that the observed multioscillatory dynamics exhibited by yeast and cardiac cells are scale-free. The results suggest a new paradigm of biological timekeeping based on fractal scaling of periodic oscillatory dynamics, characterized by a large number of frequencies as outputs on multiple time scales.

## Results

### Multi-oscillatory behavior and fractal dynamics in yeast cultures

**Figure 1:**
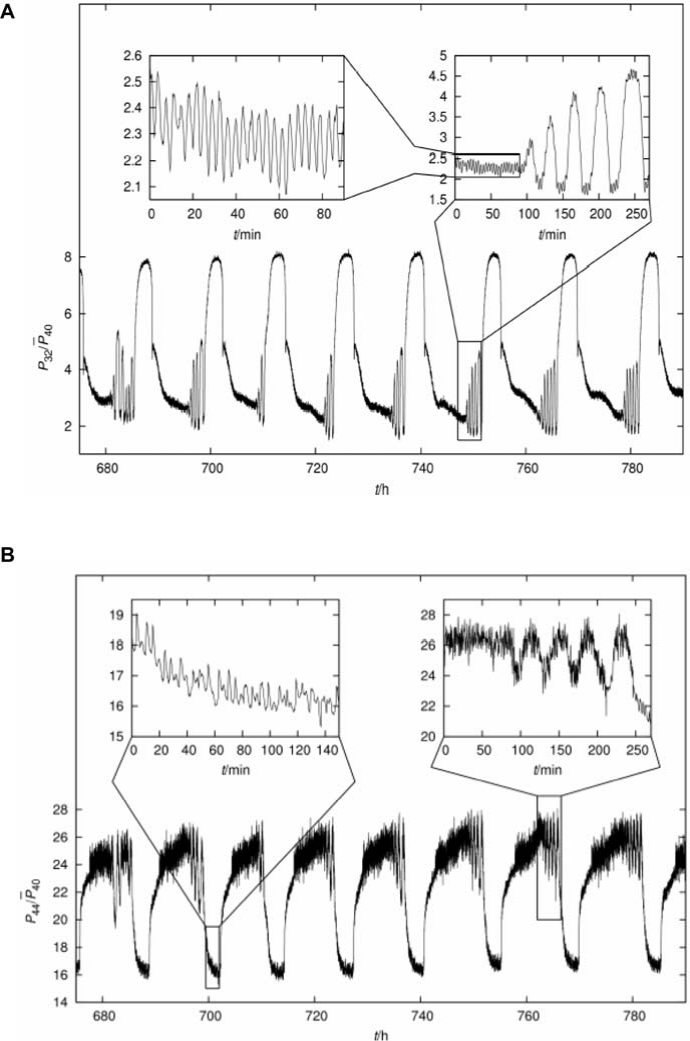
Multi-oscillatory behavior in self-organized continuous cultures of *S. cerevisiae*. Relative MIMS signals of the m/z = 32 and 44 components versus time. These mass components correspond, respectively, to O_2_ (A) and to CO_2_ (B). Time is given in hours after the start of fermentor continuous operation. The fermentors were run as described under Methods at a total volume of culture of 800 ml; medium flow rate, 1 ml per min, i.e. dilution rate D = 0.0765 h^−1^. The large-amplitude oscillation showed substantial cycle-to-cycle variability, with cycle times of 11.7 to 15.5 h, giving a mean of 13.6±1.3 h (SD, n = 8). This is longer than the mean doubling time of ln2/D∼9 h, as discussed elsewhere [35]. The biological bases for all three oscillatory outputs of the yeast culture has been confirmed by exclusion of the possible influences of variations of aeration or stirring, pulsed medium addition, cycles of NaOH addition and pH variation, or cycles of temperature control [35], [37].

It has previously been observed that yeast can produce multiple frequencies when grown continuously under precisely controlled conditions. Figure 1 shows the time course for dissolved oxygen (Fig. 1A) and carbon dioxide (Fig. 1B) concentrations from one such time series. Periods of ∼13 h, ∼40 min and ∼4 min can be detected (see the sub-panels), the lower limit is most probably the minimum sampling frequency imposed by the MIMS probe.

It has also been shown that the trajectory described by this dynamic system can be represented as a chaotic attractor [35], as the leading Lyapunov exponent was 0.752±0.004 h^−1^ (95% confidence), indicating sensitivity to initial conditions. Evidence for chaotic performance also comes from other work [36], [37], [38].

Closer examination of a part of a single long-period cycle (Fig. 1) shows the ultradian clock (∼40 min) as a super-imposed series of bursts in each dominant 13.6 h cycle. The observed variability of this rhythm [35] is probably due in part to super-positioning on the slower rhythm, although analysis of the period of ultradian clock over many cycles provides evidence for its inherently chaotic control [37]. On this time scale the CO_2_ concentration is reciprocally related to dissolved O_2_, indicating that respiration is the major process in this aerobic culture.

**Figure 2:**
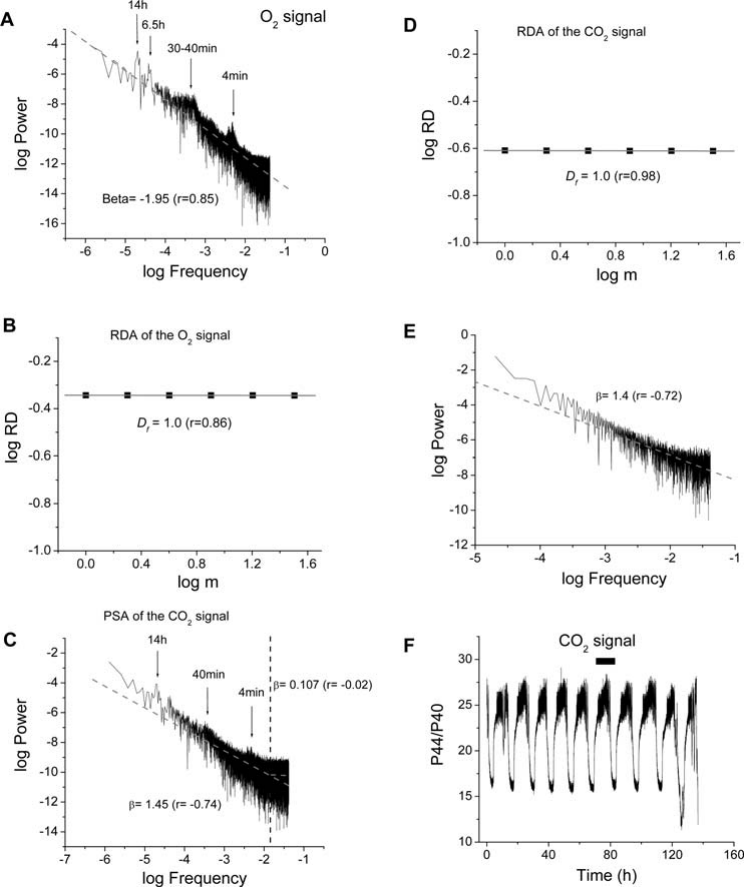
Relative Dispersional (RDA) and Power Spectral (PSA) Analysis of time series of O_2_ and CO_2_ signals obtained by MIMS from oscillating continuous cultures of *S. cerevisiae*. Both signals were processed for PSA (A, C, E) and RDA (B, D) under similar conditions as those described for heart mitochondria under Methods. The results obtained are in agreement with multiple dynamic, scale-free processes, exhibiting no predominant (characteristic) frequency. Interestingly, when the power spectrum, after Fast Fourier Transform (FFT) of the CO_2_ time series, is performed on the “hairy” top of the oscillation (panel F; the bar in the inset indicates the portion of the time series analyzed), a similar β ( = 1.4; panel E) is obtained as in the case of the low frequency (>1 min period) domain of the spectrum (β = 1.45; panel C). Clearly, the high frequency (<1 min period) domain corresponds to white noise (β∼0.0) (panel C). Panel F shows the whole CO_2_ time series analyzed.

We processed the O_2_ (Fig. 2A and 2B) and CO_2_ (Fig. 2C–E) time series (Fig. 1) using PSA and RDA. RDA revealed that the observed multioscillatory dynamics correspond to statistical fractals, as can be judged by the perfect correlation between oscillators in the 13 h, 40 min and 4 min time domains. The double log plots depicted in Figures 2B and 2D exhibit an inverse power relationship with a fractal dimension, *D_f_* ( = 1.0) implying that RD is constant with scale (i.e., the time series looks *statistically* similar at all scales) (see M&M). The inverse power law behavior is consistent with long-term memory in each of the data sets and suggests fractal dynamics of processes on different time scales (from seconds to several hours) [21], [25].

PSA also indicates an inverse power law proportional to 1/f^β^. This is as expected for a time series exhibiting self-similar scaling by RDA. The value of β = 1.95 obtained for the O_2_ signal (Fig. 2A) is close to that characteristic of colored noise, and this is again as expected for chaotic time series. The PSA of the CO_2_ signal was different as compared with the results obtained with the O_2_ power spectrum. For the CO_2_ output, we determined a value of β∼1.45 at frequencies higher than 0.016 Hz (1 min oscillatory period) whereas below this frequency only white noise was recovered, i.e. β∼0 (Fig. 2C). When we analyzed the top of the CO_2_ oscillatory response (Fig. 2F) a similar β ( = 1.4) to the whole spectrum (Fig. 2E) was obtained, arguing that this part of the signal contributes to the frequency richness of the CO_2_ power spectrum beyond the most conspicuous ones, i.e. ∼14 h, ∼40 min, ∼4 min.

**Figure 3:**
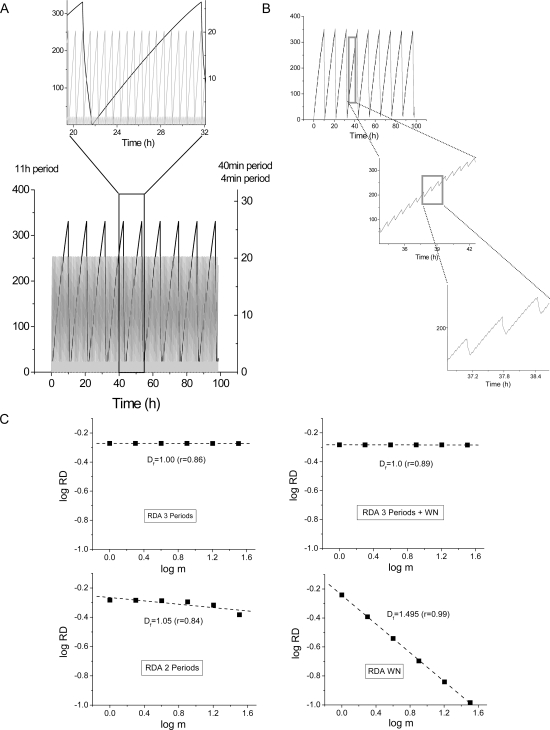
RDA of the simulated yeast time series. We simulated time series of yeast dynamics with our computational model of the mitochondrial oscillator (see M&M for details). With this model we are able to obtain oscillatory periods ranging from msec to several hours, and the parametric conditions utilized have been previously described [18], [46], [70]. Panel A shows the most conspicuous periodicities (∼11 h left *y*-axis; and 40 min and 4 min right *y*-axis) along with the fundamental trait of the inverse relationship between amplitude versus frequency (1/period) as clearly shown in the inset. Panel B depicts the unified time series after addition of the three time series shown in panel A, along with its discernible self-similarity. Panel C displays the results obtained after RDA analysis of the unified time series shown in panel B (top left); after addition of white noise (WN, top right); after taking out the longest 11 h period (bottom left). The bottom right panel in C corresponds to the RDA of WN added to the yeast time series; PSA of WN gave the expected β∼0 (not shown).

In order to further demonstrate the statistical fractal nature of the yeast dynamics we simulated a time series that captured two essential features: *i)* similarity in the periods determined experimentally; and *ii)* the inverse relationship between amplitude and frequency which is at the origin of the inverse power law determined by RDA and PSA (see Fig. 7 of [18], and its associated text about *Origins of the inverse power law behavior*). In the simulated time series (see M&M), similar to the experiment (compare Figs. 1A and 3A), we tested if: *i)* the addition of the three separate time series, corresponding to each period, into one time series allowed us to reproduce the results obtained by RDA and PSA; *ii)* there is any discernible (geometric) self-similarity in the unified time series in addition to the temporal one; *iii)* the long-term memory and statistical fractal nature of the time series is preserved when the longest 11 h period is skipped from the series; *iv)* the addition of white noise (see M&M and Fig. 3C, bottom right panel) to the unified time series abolishes or distorts the fractal dynamics. The results obtained show that the unified time series after addition of the three periods exhibits perfect correlation as determined by RDA (Fig. 3C, upper left panel), and a clearly recognizable geometric self-similarity characteristic of fractals ((Fig. 3B). This behavior was preserved after suppressing the long 11 h period (Fig. 3C, lower left panel) or adding white noise to the simulated, unified, time series (Fig. 3C, upper right panel). The PSA revealed similar results as those shown in Fig. 2A concerning the β ( = 1.92; r = 0.94) (not shown).

Taken together, these results are in agreement with the statistical fractal dynamics exhibited by the O_2_ and CO_2_ time series of yeast.

### Network dynamics of cardiac mitochondria

Mitochondria function as a source of ROS that, when kept under control, serve as important signaling molecules [39], [40], [41]. In heart, it was shown that mitochondria are organized as a network of highly correlated, coordinated, oscillators, exhibiting scale-free dynamics in ΔΨ_m_ [18], [42]. Since the coordination between mitochondria within the network appears to be ROS-mediated, it was important to know whether these crucial signaling molecules also exhibited scale-free dynamics.

**Figure 4:**
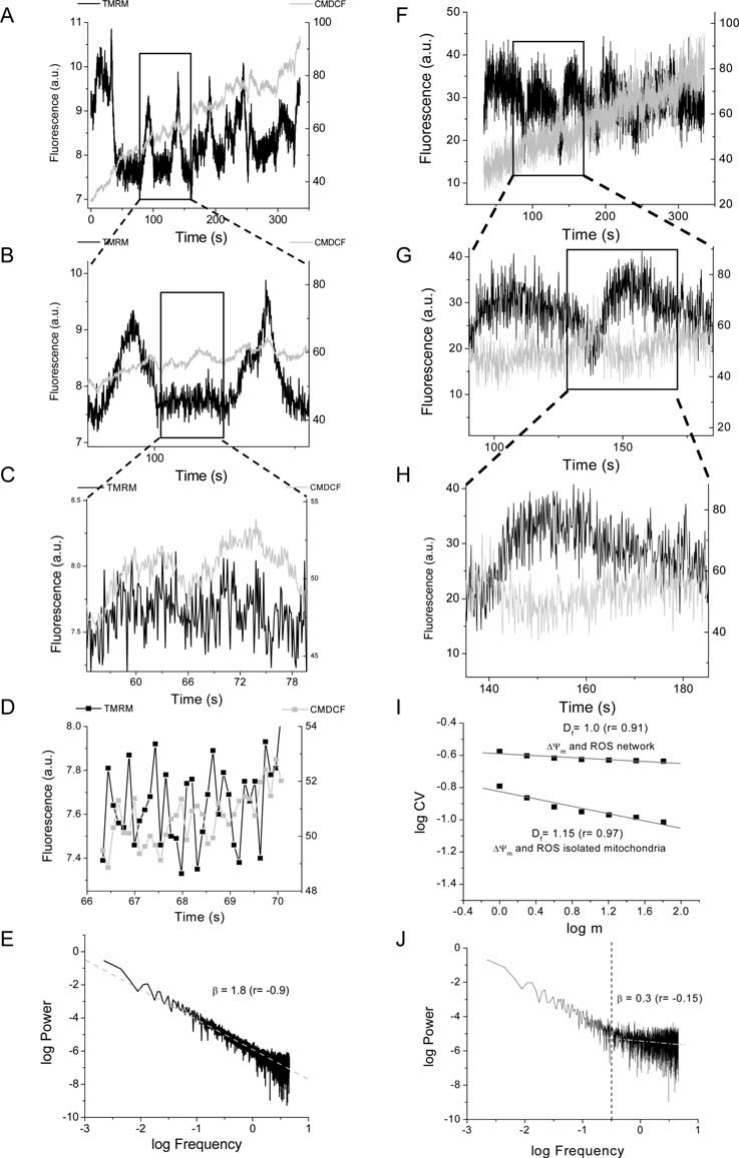
RDA and PSA of ΔΨ_m_ and Reactive Oxygen Species (ROS) fluorescence time series from a mitochondrion or the mitochondrial network of cardiomyocytes. Time series of the mitochondrial network within the oscillating mitochondrial cluster (A–D) or of a mitochondrion outside the cluster (F–H) were analyzed in freshly isolated ventricular cardiomyocytes loaded with 100 nM TMRM, a ΔΨ_m_ sensor, and 2 µM CMH_2_DCF, a ROS sensor, and imaged by two photon microscopy (110 ms time resolution). The results obtained from a stack of 3,050 images are shown. A) This panel shows the time series from the mitochondrial network of a cardiomyocyte loaded with TMRM and CMDCF and imaged as described above. In panels A–C and F–H, the ΔΨ_m_ and ROS signals from the mitochondrial network (A–C) or an isolated mitochondrion (F–H) are depicted at increasing degrees of magnification; notice the degree of self-similarity of both signals. The latter was confirmed by PSA (E) and RDA (I) analyses of the time series. Panel D shows the expected phase relationship between the TMRM and CM-DCF signals due to Fluorescence Resonance Energy Transfer between both fluorophores [43]. E, J) The time series of TMRM or CM-DCF fluorescence was subjected to FFT as described in the Methods section. The power spectrum was obtained from the FFT of the TMRM or CM-DCF signal as the double log plot of the amplitude (power) *versus* the frequency. This relationship obeys a homogeneous power law (1/ *f* ^β^; with *f*, frequency, and β, the spectral exponent) and is statistically self-similar which means that there is no dominant frequency. The PSA reveals a broad spectrum of oscillation in normally polarized mitochondria with a spectral exponent of β = 1.79 while a random process (white noise) gives a β∼0 meaning that there is no relationship between the amplitude and the frequency in a random signal. A β = 1.0 or 2.0 corresponds to pink or brown noise, respectively. The inverse power law spectrum arises from the coupling of frequency and amplitude in an orderly statistical sequence. I) RDA: A log-log plot of the relative dispersion, RD ( = SD/mean), of the fluorescence distribution obtained at increasing values of the aggregation parameter, *m* (see also the Methods section) gives a fractal dimension, D_f_, close to 1.0, under “physiological” conditions (panel I, top trace). A completely random process gives D_f_ = 1.5. The data obtained from RDA was subjected to linear regression and the slope calculated (D_f_ = 1 –slope). Randomization of the time series of the TMRM fluorescent signal gives a value of β close to zero similar to the spectrum shown in panel J, as opposed to a β = 1.8 in the non-randomized signal obtained from the mitochondrial network (E). The spectral exponent β = 1.8 (E) is consistent with long-range correlations that, after signal randomization becomes white noise, with loss of correlation properties β = 0.3 (≅0) (J).

Here, we analyze simultaneously the ΔΨ_m_ or NADH and ROS time series of cardiomyocytes by two photon microscopy with high (∼100 ms) temporal resolution. ROS were probed with two different sensors, CMH_2_DCF, for H_2_O_2_ [43] and MitoSox, for superoxide free radical, O_2_^.−^ [44]. CMH_2_DCF was assayed in parallel with the ΔΨ_m_ sensor, while MitoSox was monitored together with the cell's autofluorescence. Both, CMH_2_DCF and MitoSox exhibit scale-free dynamic behavior, as expected from a network of coupled oscillators [18]. Shown in Figure 4 are the time series of ΔΨ_m_ and the H_2_O_2_ sensor signals from a mitochondrial network (Fig. 4 A–E) or an individual mitochondrion (Fig. 4 F–J). By RDA and PSA (Fig. 4 A–C, and F–H) we show that mitochondrial dynamics in cardiomyocytes also exhibit, as in the evolutionarily distant yeast, a fractal behavior of their temporal organization. Both ΔΨ_m_ and ROS signals are highly correlated (Fig. 4A–C) according to PSA (Fig. 4E) and RDA (Fig. 4I, top trace). This correlation decreases in isolated mitochondria (Fig. 4F–H) as shown by increased white noise behavior in the power spectrum (Fig. 4J) and lower correlation by RDA (Fig. 4I, lower trace). Figure 4D shows that in the millisecond time scale (*ca.* 450 ms period) both signals exhibit the correct phase relationship [43].

**Figure 5:**
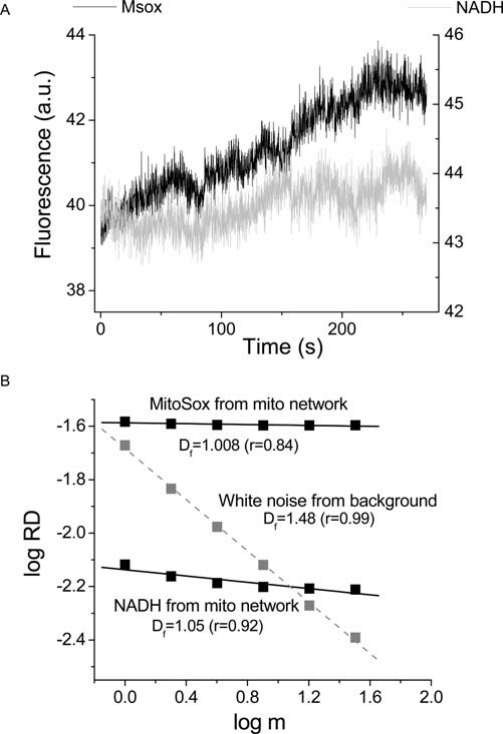
RDA of ROS and NADH fluorescence time series from the mitochondrial network of cardiomyocytes. Freshly isolated cardiomyocytes were loaded with 2 µM MitoSox for at least 20 min at 37°C, and imaged by two photon microscopy (120 ms time resolution). A) Shown is a representative result for MitoSox and NADH fluorescence obtained from a stack of 2,200 frames. Notice the ladder-like increase in the MitoSox signal with each staircase corresponding to membrane potential depolarization and NADH oxidation which is concomitant with a burst of superoxide production (Zhou, Aon and O'Rourke, submitted). Panel B shows the results of the RDA analysis as applied to the time series shown in panel A.

The statistical fractal nature of the ROS signal was further confirmed by RDA utilizing the sensor of O_2_^.−^ MitoSox, the ROS species that our experimental and theoretical evidence favors as the intracellular messenger between mitochondria [43], [45], [46] (Fig. 5). In this case the ROS signal was analyzed concomitantly with the cell's autofluorescence. Both signals behaved as statistical fractals (Fig. 5B). The MitoSox fluorescence should increase in a stepwise fashion during mitochondrial oscillations (Fig. 5A). In both signals, the fractal dimension, *D_f_*∼1.0, obtained after RDA is clearly different from uncorrelated white noise obtained from the image background (Fig. 5B). This result is consistent with long-term memory in agreement with the statistical fractal dynamics of the ROS and NADH signals.

### Mechanistic insights into the conserved core of the scale-free dynamics exhibited by yeast and heart

The inverse power law behavior of the power spectrum and the invariant RD across temporal scales obtained from the analysis of time series in yeast and cardiac mitochondria are hallmarks of scale-free dynamics, i.e. multiple oscillatory frequencies in a wide range of time scales spanning at least three orders of magnitude.

**Figure 6:**
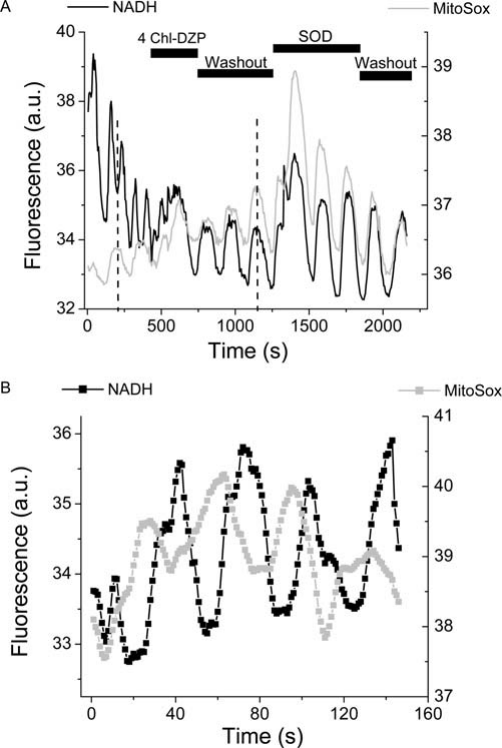
Yeast synchronous oscillations of NAD(P)H in the minute time scale are blocked by an inhibitor of the IMAC. A) The whole cell NAD(P)H and MitoSox oscillations were obtained from a microscopic field of ∼30 yeast perfused with aerated PBS, pH 5.0, in the presence of 5 mM glucose under the conditions specified in Methods. When indicated, 32 µM of 4 Chl-DZP or 40 µg/ml of SOD were acutely added to the chamber and washed out. The average fluorescence from the whole microscopic field was collected at 605±25 nm (MitoSox) or at <490 nm (NAD(P)H). B) Shown is the phase relationship between the NAD(P)H and MitoSox signals from a control experiment in which the drugs were not administered. The dashed lines in panel A are for indicating the phase relationship between the NAD(P)H and MitoSox signals.

We next asked whether, in both species, similar underlying mechanisms were at play in the scale-free dynamics observed (see Supplementary movie S1 and S2 of cardiomyocytes and yeast NAD(P)H oscillations in the minute time scale, respectively). A key mechanistic insight was obtained when yeast oscillations of NAD(P)H and ROS in the minute time scale could be reversibly suppressed with 4-chlorodiazepam (4 Chl-DZP) (Fig. 6), the same mitochondrial inner membrane anion channel blocker of the oscillations in heart cells [43]. More precisely, we studied whether ROS (specifically O_2_^.−^) and IMAC were involved in the oscillatory (as in heart), and synchrony mechanisms observed in yeast at the subcellular and cellular levels in the minute time scale [47]. Figure 6 shows that the spontaneous NAD(P)H oscillations exhibited by the same yeast strain utilized in the continuous cultures (Fig. 1) could be suppressed by 4 Chl-DZP, an antagonist of the mitochondrial benzodiazepine receptor, and IMAC blocker [43]. The NAD(P)H oscillations reappeared after 4 Chl-DZP washout, and persisted even after addition of superoxide dismutase (SOD), a O_2_^.−^ scavenger, to the medium (Fig. 6A). Importantly, the O_2_^.−^ sensor MitoSox oscillated out of phase with the yeast autofluorescence (Fig. 6B) as expected from a large increase in ROS production and release from mitochondria after NAD(P)H oxidation, in phase with ΔΨ_m_ depolarization [47]. Since MitoSox is irreversibly photo-oxidized by ROS, one would have expected it to increase in a stepwise fashion as in cardiomyocytes (see Fig. 5A). However, the decrease in signal observed in yeast suggests that the massive burst of intracellular O_2_^.−^ production is followed by its release to the extracellular medium, including photo-oxidized MitoSox whose fluorescence decreases by dilution. Interestingly, after 4 Chl-DZP treatment and washout, the NAD(P)H and ROS signals started to oscillate in phase. The latter observation together with SOD's inability to block the oscillations, suggest that O_2_^.−^ contributes to intra- rather than inter-cellular signaling. As time elapses, this signaling effect becomes more prominent thus explaining the increase in amplitude of both oscillations and the *apparent* in phase relationship between NAD(P)H and ROS (Fig. 6A).

## Discussion

We have been able to show that the multi-oscillatory behavior of yeast and heart cells corresponds to statistical fractal dynamics. This behavior is consistent with scale-free dynamics spanning a wide range of frequencies of at least three orders of magnitude. The significance of scale-free temporal organization for organelle, cell, and organism timekeeping cannot be overstated as, potentially, what affects one time scale affects them all: a fundamental property of dynamic fractals [25], [48], [49]. To our knowledge, this is the first formal description of such dynamics in two evolutionary distant eukaryotic systems.

Both yeast cells and heart mitochondria behave as networks of coupled oscillators. In eukaryotes, mitochondria act as metabolic “hubs” [42] producing ROS as signaling molecules with scale-free dynamics (Figs. 4 and 5). Moreover, the oscillatory dynamics from both yeast and heart are temperature-compensated, supporting the role of these oscillators as biological clocks or timekeepers [49], [50].

In cardiomyocytes each mitochondrion functions as an autonomous oscillator [46] coupled through ROS to its neighbors [18], [42]. The resulting temporal coordination is weak under physiological conditions where the dynamic is characterized by an inverse power law in frequency, spanning at least three orders of magnitude. However, under pathological conditions, such as reperfusion after ischemic injury, the mitochondrial coordination becomes strong and the frequency spectrum narrows dramatically to low frequency, large amplitude oscillations [18], [29].

In yeast cell populations, individual mitochondria, as well as single cells, oscillate autonomously during spontaneous periodic behavior on the minute time scale [47]. Respiratory oscillations in yeast (τ∼40 min) involve an intracellular network of interactions that embraces metabolic, transcriptional, mitochondrial, and cell division cycle processes and their control systems; key effectors include H_2_S [51], acetaldehyde [52], and ROS [53]. Our data indicate that the respiratory activity of yeast also shows fractal scaling with multiple outputs in different time scales across at least 3 orders of magnitude. Further inspection of the power spectrum of dissolved CO_2_ signal revealed low (hours), and high (minutes) frequency domains. This bi-domain behavior arises mainly from the accumulation of points in the high frequency domain (periods of a few minutes), due to small period oscillations riding on top of the CO_2_ signal (which also exhibits the conspicuous frequencies seen in the O_2_ signal, marked with arrows in Fig. 2C). Since the amplitude of the signal is much higher than the “noise” that stems from the equipment [35] these are also oscillations of biological origin. Unlike O_2_, CO_2_ is an *output* of the growing yeast population implying that signaling processes (i.e. secreted products of the yeast metabolic system) in the high frequency domain are relevant for keeping the oscillators synchronized, i.e., when respiration is maximal.

### Scale-free dynamics from scale-free networks? Experimental and theoretical precedents from the cardiac mitochondrial network

In the present work, the time series analyzed express the collective dynamics exhibited by mitochondria at the subcellular (heart) and cellular as well as cell population levels (yeast) resulting in emergent self-organized spatiotemporal behavior under the conditions analyzed. Whether the resulting scale-free dynamics exhibited by both cellular systems stems from scale-free networks is a main question raised by our findings.

From a biological standpoint, three main approaches have characterized the study of cellular networks (reviewed in [42]): *i)* architectural (structural morphology), *ii)* topological (connectivity properties), and *iii)* dynamical. Within this framework, network can refer to spatial (structural and topological) as well as temporal (different dynamics) aspects of metabolism, and more specifically, mitochondrial energetics. In the cellular realm, *dynamic organization* [6] encompasses the architectural and the topological views of network analysis, accounting for both the autonomous dynamics exhibited by their components (nodes) and their defined interactions (connectivity) based on kinetic and thermodynamic principles [42], [48]. As such, our vision of networks includes the approach based on graph theory which emphasizes the topological aspects of network connectivity [23], [24].

Morphologically, mitochondria form regular lattice-like networks as in heart cells, or irregular, filamentous structures, as in yeast [47], neuron or cancer cells (reviewed in [42]). Biochemically, by being poised at the convergence of most anabolic and catabolic pathways, through the tricarboxylic acid cycle, mitochondria represent true metabolic “hubs” due to their multiple links to other pathways as either an input (source) or an output (sink). Dynamically, the idea that mitochondria may function as a coordinated network of oscillators emerged from studies in living cardiomyocytes subjected to metabolic stress [43], [54]. The network behavior of mitochondria depends on local as well as global coordination in the cell [45], ROS-induced ROS release [55] is the mechanism that was recently shown to exert both local and cell-wide influence on the network [43], [45].

Theoretical simulations indicated that the mitochondrial oscillator's period can be modulated over a wide range of time scales [46] and together with the fact that the period of the oscillations is temperature-compensated within a 12°C range (25°C to 37°C), suggested that the mitochondrial oscillator may be an intracellular timekeeper with the characteristics of a biological clock [49]; the latter trait being shared with yeast [50]. The theoretical feasibility of this proposal was anticipated by the work of Winfree (1967) [56], Kuramoto (1984) [57], and Strogatz (2000) [58], among others (see [59] for a review), when they addressed the problem of how hundreds or thousands of coupled oscillators achieve synchrony. A main finding arising from those studies was that synchronization occurs cooperatively from an initial nucleus where a few oscillators happen to sync and then recruit other oscillators, making the initial nucleus even larger and amplifying its signal [59]. After the initial nucleus achieves a threshold given by a critical mass of oscillators in phase, the population spontaneously self-synchronizes as in a phase transition. We also observed, as Winfree and Kuramoto had, the analogue of a phase transition at the turning point between the physiological and pathophysiological regimes in the mitochondrial network (see Fig. 1 in [18]). Experimentally, this global phase transition (visualized as a cell-wide mitochondrial depolarization involving a coordinated response of at least 60% of the mitochondrial population) is attained when a critical density of mitochondria accumulates ROS above a threshold to form an extended spanning cluster [45]. In fact, the spanning cluster in our work may be considered analogous to the nucleus of synchronized oscillators as described in the work of Winfree and Kuramoto [59]. We coined the term “mitochondrial criticality” to refer to the state of the system just before network depolarization [30], [45]. These results are in good agreement with the quantitative predictions derived from percolation theory, especially concerning the percolation threshold [45], the fractal (spatial) organization exhibited by percolation processes at the threshold [60], and the critical exponents [45].

At this stage, our theoretical work in heart cells is consistent with either of two possibilities: *i)* mitochondrial dynamics becomes rapidly unstable and traverses a Hopf bifurcation after which pronounced oscillations occur in all of the energetic state variables (i.e., ΔΨ_m_, redox potential, ATP∶ADP ratio, VO_2_, etc.) [46] or *ii)* the mitochondrial network dynamics, already in the oscillatory domain under physiological conditions, locks into a low-frequency high amplitude oscillation through strong synchronization mediated by a ROS-induced ROS release mechanism [30], [43]. At present, our experimental work favors the second possibility.

The finding that cardiac mitochondria lock to a dominant frequency and high amplitude ΔΨ_m_ oscillation under pathological conditions (e.g., ischemia–reperfusion) as a self-organized phase transition, raises parallels between the mitochondrial network and many other physical, chemical, and engineered systems [42], [45], [48]. Systems of disparate nature, when subjected to excessive loads, approach a critical state at which they become extremely sensitive to perturbations that can be efficiently propagated under these conditions [61], [62]. Because of the intrinsic nonlinear properties of mitochondria, new emergent macroscopic behavior appears, including spatiotemporal synchronization visualized as oscillations in energetics and waves of ΔΨ_m_ depolarization [45].

### The mitochondrial network and thiols redox cycling are at the core of the yeast and heart rhythmicity

**Figure 7:**
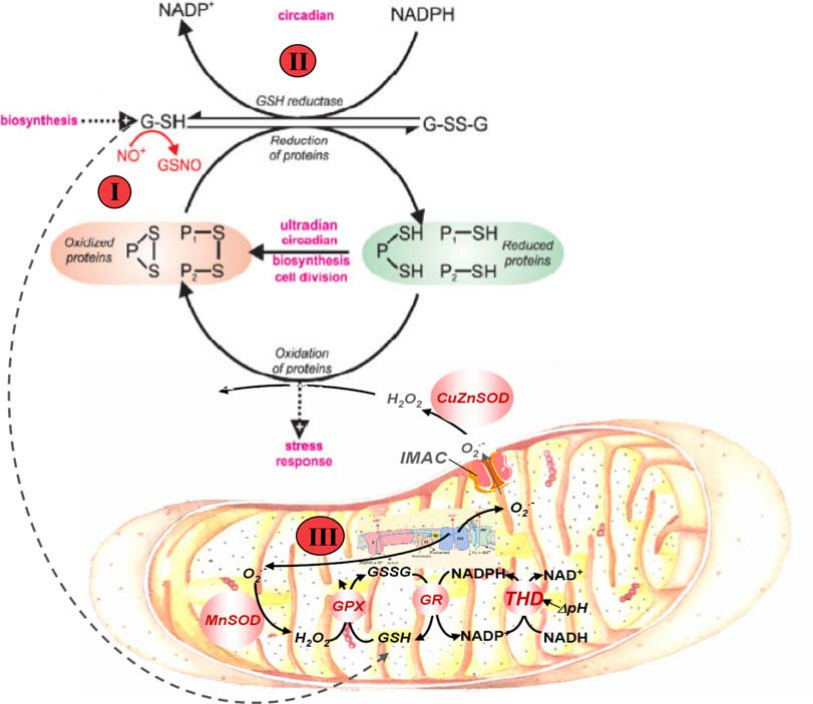
Redox cycling of intracellular thiols at the core of rhythmicity. The scheme shows that generation of rhythms entails the cycling of cytoplasmic and mitochondrial proteins between their oxidized and reduced states mainly driven by ROS and the redox potential of the thiols pool. Mitochondria are the main source of ROS produced by the respiratory chain; oxidative stress results from an imbalance between ROS production and ROS scavenging. The glutathione redox potential, and the absolute concentrations of reduced (GSH) and oxidized (GSSG) glutathione, modulate ROS production in mitochondria. GSH regeneration in the mitochondrial matrix is essential for keeping the ROS balance namely through glutathione reductase (GR) and transhydrogenase (THD). Although yeast has not had any THD activity confirmed, thioredoxin and glutaredoxin may play a crucial role. In turn, the redox status in the mitochondrial matrix represented by NADH and NADPH pools, which are interconverted by transhydrogenase activity, drives GSH regeneration. Mitochondrial GSH is also replenished via cytoplasmic import through carriers. In cardiomyocytes, mitochondrial oscillations are triggered when a threshold level of ROS is attained [43] which happens when the glutathione redox potential oxidizes to GSHz∶GSSG ratios between 150∶1 to 100∶1. A critical ratio of GSH∶GSSG of 50∶1 elicits the opening of the permeability transition pore, cell hypercontraction, and death [71]. In yeast, numerous processes (magenta I–III) have proven to be oscillatory and we propose that ensembles of oscillators are coupled via this primordial mechanism. In yeast, these processes may be modulated by illumination, temperature changes or chemical perturbation. Perturbation analysis of the yeast ultradian system utilising NO^+^ donors [72], 5-nitro-2-furaldehyde (I), D,L-butathionine (S,R)-sulphoximine (II) [73] or protonophores (e.g. carbonyl cyanide m-chlorophenylhydrazone) (III) [74], confirms the central role of this redox system; the numbers on the figure represent the site of perturbation.

At the core of the scale-free dynamics in yeast and heart lies a redox cycle that involves NAD(P)H, glutathione and protein-thiols [11], [12], [63] (Fig. 7), and a more detailed overview has recently been formulated [13]. In heart, the redox cycling involves at least glutathione, and NAD(P)H couples in the mitochondrial matrix, as modulators of ROS generation. Importantly, the cellular redox potential, represented by the GSH/GSSG and NAD(P)H/NAD(P)^+^ ratios, and the absolute concentration of the species, determine when ROS production will accelerate by the activation of inner mitochondrial membrane ion channels (inner membrane anion channel, IMAC, and permeability transition pore, PTP). The sequential opening of mitochondrial channels elicits ROS production that at threshold levels of glutathione redox potential triggers a transition between the physiological and pathophysiological regimes of cardiac mitochondria network dynamics [18], [30].

A crucial test of whether yeast and heart cells share a common mechanism at the origin of the oscillatory dynamics (Fig. 7) was given by the ability of 4 Chl-DZP (Ro5-4864) to block the spontaneous 1–2 min period oscillations exhibited by yeast (Fig. 6). The significance of this result is further strengthened when one considers that oscillations in yeast reflect synchronous oscillations of mitochondria at the subcellular level, as recently demonstrated [47]. The direct involvement of O_2_^.−^ and IMAC in the yeast oscillations brings about a direct mechanistic relationship with mitochondrial oscillations in cardiomyocytes, at least in the minute time scale.

As a specific antagonist ligand of the peripheral benzodiazepine receptor (PBR) located in the mitochondrial membrane, 4 Chl-DZP blocks the IMAC along with the oscillations in cardiomyocytes [43]. In an ischemia-reperfusion scenario, 4 Chl-DZP was able to protect whole hearts from reperfusion-related arrhythmias [29] and helped to preserve mechanical function [64] after ischemic injury. Our results are in agreement with those reported in the literature showing that mitochondria from the yeast *S. cerevisiae* as well as the amoeba *Acanthamoeba castellanii* contain proteins able to bind Ro5-4864 (i.e. a synonym for 4 Chl-DZP) with affinity comparable to rat liver mitochondria [65]. Apparently, the interaction with the voltage dependent anion selective channel (VDAC) and the adenine nucleotide translocase (ANT) is required by the 18 Kda polypeptide constituting PBR to interact with 4 Chl-DZP in yeast [66].

### Concluding Remarks

The present and recent contributions show that mitochondria oscillate as coupled oscillators either during respiratory oscillations in yeast or in cardiac mitochondria. It is now clear that yeast or mitochondrial populations function like a network of coupled oscillators, through chemical communication by metabolites. The demonstration of the existence of self-organization, scaling, criticality, percolation, and fractals in the cardiac mitochondrial network shows that there exists a clear crossroad between the universality of physical concepts and crucial (patho)physiological functions in the heart. The multiple time scales exhibited by two evolutionary distant systems such as heart and yeast suggest that intracellular network dynamic organization manifests itself as scale-free in the form of dynamic fractals. The scale-free behavior exhibited by mitochondrial network dynamics would allow modulation of intracellular timekeeping in several time scales simultaneously. For the coherent organization of the cellular networks of metabolism, biosynthesis, assembly processes and cell cycle progression, correlated oscillatory function on multiple time scales is a characteristic of the living state.

## Materials and Methods

### Cardiomyocyte isolation

In accordance with *Guide for the Care and Use of Laboratory Animals* (NIH, No. 85-23, 1996) and the Johns Hopkins Animal Care and Use Committee, adult guinea pigs (300 g) were anesthetized with 260 mg pentobarbital and 1000 U heparin sodium (i.p.). The hearts were then excised and subjected to the procedure of isolation of ventricular myocytes by enzymatic dispersion as previously described [67]. All experiments were carried out at 37°C on freshly isolated isolated cardiomyocytes.

After isolation, cells were stored briefly in a high K^+^ solution (in mM: 120 potassium glutamate, 25 KCl, 1 MgCl_2_, 10 HEPES, 1 EGTA, pH 7.2 with KOH) and either used immediately or transferred to Dulbecco's Modification of Eagle's Medium (10-013 DMEM, Mediatech, Inc. Virginia) in laminin-coated petri dishes in a 95% O_2_, 5% CO_2_ incubator at 37°C for at least 2 h before imaging. As previously described [18], [43], experimental recordings started after exchange of the DMEM with Tyrode's solution containing (in mM): 140 NaCl, 5 KCl, 1 MgCl_2_, 10 HEPES, 1 CaCl_2_, pH 7.4 (adjusted with NaOH), supplemented with 10 mM glucose. The dish containing the cardiomyocytes was equilibrated at 37°C with unrestricted access to atmospheric oxygen on the stage of a Nikon E600FN upright microscope.

### Fluorescent probes for two-photon laser scanning microscopy and image acquisition and analysis

The cationic potentiometric fluorescent dye TMRM (100 nM) and the fluorescent probe 5-(–6)-chloromethyl-2′,7′-dichlorohydrofluorescein diacetate (2 µM CM-H_2_DCFDA, Invitrogen-Molecular Probes, Eugene, OR) were used to monitor changes in ΔΨ_m_ and ROS, respectively [43]. Images were recorded using a two photon laser scanning microscope (Bio-Rad MRC-1024MP) with excitation at 740 nm. The red emission of TMRM was collected at 605±25 nm, and the green emission of CM-DCF was recorded at 525±25 nm (Tsunami Ti∶Sa laser, Spectra-Physics).

### Yeast culture oscillation data and two photon imaging of yeast oscillations

We reanalyze a data series originally described elsewhere [35]. Briefly, an autonomously oscillating culture of *S. cerevisiae* under constant environmental conditions (temperature, illumination, pH) was monitored by membrane-inlet mass spectrometry [68]. Data were collected every 12 s at m/z = 32, 34, 40 and 44 corresponding to oxygen, H_2_S, argon and carbon dioxide. Argon m/z = 40 was used to correct for long-term drift in the instrument's response as described previously [35].

Spontaneous, synchronized oscillations in a contiguous layer of *S. cerevisiae* cells loaded with 2 µM MitoSox (Invitrogen-Molecular Probes, Eugene, OR) incubated at 30°C with aeration of the perfusion buffer, were monitored by two-photon scanning laser microscopy. Yeasts were attached to a coverslip which had been coated with poly-L-lysine with unrestricted access to atmospheric oxygen on the stage of a Nikon E600FN upright microscope which was maintained at 30°C [47].

### Analysis of time series from yeast and heart systems

Extended time series were obtained from isolated cardiomyocytes loaded with TMRM, a ΔΨ_m_ probe, or either of two different ROS probes, CMH_2_-DCF and MitoSox (H_2_O_2_ or superoxide, O_2_^.−^, sensor, respectively). In cardiomyocytes the time series of the fluorescencent probes, consisting of 2000 to 4000 images with temporal resolution of 110 ms to 120 ms, were performed with the couples TMRM-CMH_2_DCF or MitoSox-NADH. The yeast time series of O_2_ and CO_2_ (47,200 time points with temporal resolution of 12 s representing 118 h of continuous culture) were recorded simultaneously from yeast cultures, and subjected to Relative Dispersional Analysis (RDA) and Power Spectral Analysis (PSA).

RDA provides a quantitative measure of how the state of a process at a given time point is influenced by the state of the system at previous time points [21], [25], [69]. The RD is repeatedly calculated while binning (coarse-graining) the data set at successively larger time-scales. Aggregation of adjacent points in the time series at 2,4,8,16 and 32 successive values of the data set to calculate the RD was performed for each grouping [25] and this was plotted against the aggregation number, m. The slope of this relation provides information on the extent of long-term correlation (memory) as well as the statistical fractal nature of the dynamics in the time series.

PSA: The power spectrum of the time series was analyzed after Fast Fourier Transform. Double log plots of amplitude versus frequency indicated a decrease of power proportional to 1/f^β^, where f is frequency and β is the spectral exponent.

### Computational simulation of the yeast time series

Oscillations with similar periods to those exhibited by yeast (see Fig. 1) were reproduced utilizing our mathematical model of the mitochondrial oscillator, where we are able to vary the oscillatory period from msec to several hours [18], [46], [49]. The ROS-dependent mitochondrial oscillator of cardiac cells has been described experimentally and theoretically. This computational model incorporates mitochondrial ROS production, ROS scavenging, and inner membrane anion channels (IMAC) into a previously developed model of cardiac mitochondrial energetics and Ca^2+^ dynamics [46], [70]. The mitochondrial oscillator model has been validated by extensive simulation of reported experimental evidence, and experimental verification of specific model predictions [18], [46].

The time series for each period contained 354,000 data points (sampled every 20 s) spanning ∼100 h as in the yeast synchronous culture (Fig. 1). Each one of the time series exhibiting 11 h, 40 min, or 4 min oscillation period was simulated with the same (fixed) integration step to avoid aliasing effects.

White noise (WN) was simulated with a random number generator for a similar time period as the surrogate oscillatory time series, and the expected characteristics of WN were confirmed by RDA and PSA, obtaining *D_f_*∼1.5 and β∼0, respectively.

## Supporting Information

Movie S1 Cardiomyocyte NAD(P)H oscillations. Movie of NAD(P)H oscillations (autofluorescence) in a cardiomyocyte recorded using a two photon laser scanning microscope (Bio-Rad MRC-1024MP) with excitation at 740 nm. Whole cell oscillations (100 s period) were triggered with a laser flash in an isolated cardiomyocyte in the absence of any other fluorophore. Notice that the NAD(P)H signal is coming mostly from mitochondria (seen as a lattice), thus the oscillations correspond to the mitochondrial network. Bar, 10 µm
(14.61 MB AVI)

Movie S2 Yeast NAD(P)H oscillations. Movie of NAD(P)H oscillations (autofluorescence) in spontaneously synchronized oscillations (ca. 100 s) in a contiguous layer of S. cerevisiae cells, recorded with a two photon laser scanning microscope (Bio-Rad MRC-1024MP) with excitation at 740 nm. The layer of yeast cells was perfused with aerated PBS, pH 7.4, in the presence of 5 mM glucose. The oscillations shown in the video correspond to single cell as well as synchronous oscillations of mitochondria at the subcellular level. This particular video has also been supplementary material in Aon et al., 2007. Bar, 3 µm.
(15.31 MB AVI)
